# Abigene, a Prospective, Multicentric Study of Abiraterone Acetate Pharmacogenetics in Metastatic Castration-Resistant Prostate Cancer †

**DOI:** 10.3390/pharmaceutics15020651

**Published:** 2023-02-15

**Authors:** Jean-Marc Ferrero, Hakim Mahammedi, Gwenaelle Gravis, Guilhem Roubaud, Philippe Beuzeboc, Remi Largillier, Delphine Borchiellini, Claude Linassier, Nathalie Ebran, Tanguy Pace-Loscos, Marie-Christine Etienne-Grimaldi, Renaud Schiappa, Jocelyn Gal, Gérard Milano

**Affiliations:** 1 Medical Oncology Department, Centre Antoine Lacassagne, University Côte d’Azur, 06189 Nice, France; 2 Medical Oncology Department, Centre Jean Perrin, 63011 Clermond Ferrand, France; 3 Medical Oncology Department, Institut Paoli Calmette, 13009 Marseille, France; 4 Department of Medical Oncology, Institut Bergonié, 33076 Bordeaux, France; 5 Medical Oncology Department, Institut Curie, 75005 Paris, France; 6 Medical Oncology Department, Centre Azuréen de Cancérologie, 06250 Mougins, France; 7 Medical Oncology Department, Centre Hospitalier Régional Universitaire, 37044 Tours, France; 8 Oncopharmacology Unit, Centre Antoine Lacassagne, University Côte d’Azur, 06189 Nice, France; 9 Epidemiology and Biostatistics Department, Centre Antoine Lacassagne, University Côte d’Azur, 06189 Nice, France

**Keywords:** prostate cancer, abiraterone acetate, pharmacodynamics, pharmacogenetics

## Abstract

Abiraterone acetate (AA) is the first-in-class of drugs belonging to the second-generation of agents inhibiting androgen neosynthesis in advanced prostate cancer. A cumulative experience attests that germinal gene polymorphisms may play a role in the prediction of anticancer agent pharmacodynamics variability. In the present prospective, multicentric study, gene polymorphisms of CYP17A1 (AA direct target) and the androgen transporter genes SLCO2B1 and SLCO1B3 (potential modulators of AA activity) were confronted with AA pharmacodynamics (treatment response and toxicity) in a group of 137 advanced prostate cancer patients treated in the first line by AA. The median follow-up was 56.3 months (95% CI [52.5–61]). From multivariate analysis, rs2486758 C/C (*CYP17A1*) and PSA (≥10 ng/mL) were associated with a shorter 3-year biological PFS (HR = 4.05, IC95% [1.46–11.22]; *p* = 0.007 and HR = 2.08, IC95% [1.31–3.30]; *p* = 0.002, respectively). From a multivariate analysis, the rs743572 (*CYP17A1*) and performance status were independently associated with significant toxicity (OR = 3.78 (IC95% [1.42–9.75]; *p* = 0.006 and OR = 4.54; IC95% [1.46–13.61]; *p* = 0.007, respectively). Host genome characteristics may help to predict AA treatment efficacy and identify patients at risk for toxicity.

## 1. Introduction

The next generation of hormonal agents has improved treatment outcomes in patients with metastatic castration-resistant prostate cancer (mCRPC). Abiraterone acetate (AA) is the first drug developed in this class of second-generation androgen biosynthesis inhibitors [1]. Moreover, although AA has demonstrated a favorable safety profile, it is not without severe side effects related to its mechanism of action. No objective individual criteria currently exist for identifying either suitable candidates or very bad responders to AA treatment. In this setting, the clinical relevance of potential predictive factors remains to be established in order to orientate patients to the most adapted treatment after the failure of androgen deprivation therapy in mCRPC.

It is accepted that tumor genomics may offer interesting possibilities to personalize treatment. However, besides somatic genomics, the patient’s genome characteristics also play a role. The global purpose of pharmacogenetics is thus to try to put into evidence the links between individual gene characteristics and treatment pharmacodynamics in terms of response and toxicity. A suitable example of clinically validated application of the pharmacogenetics of anticancer agents is the individual UGT1A1 polymorphism knowledge permitting a more secure use of irinotecan [2]. The area of germinal gene polymorphisms playing a potential role in the pharmacodynamics variability of anticancer agents covers more generally a wide range of compounds, including cytotoxic drugs and kinase inhibitors [3]. Globally, pharmacogenetics-related studies point to the importance of single nucleotide polymorphisms (SNPs) in drug targets and drug-metabolizing enzymes for the effects of anticancer drugs.

It has been shown that testosterone levels within metastatic tumoral tissue from men receiving hormone therapy were significantly higher than those from primitive tumors of untreated prostate cancers. Among the mechanistic explanations for this observation, it has been shown that CYP17A1, a key enzyme in de novo steroid synthesis localized in the testis and adrenal gland, is up-regulated in CRPC metastases [4,5]. AA is an irreversible inhibitor of CYP17A1. This enzyme controls androgen production at the tumor level. The CYP17A1 gene presents numerous SNPs. A functional impact of these mutations has been suggested for nine of them, and they have been previously shown to impact, at variable degrees, the development of prostate cancer and to influence prostate cancer patients’ survival [6]. Considering the AA mechanism of action, one can make the hypothesis that point mutations altering CYP17A1 function and/or expression could directly impact AA antitumor activity by modulating the levels of the natural androgen competitor. The presence of SNPs in genes involved in the membrane transport of testosterone and dehydroepiandrosterone (DHEA), namely SLCO2B1 and SLCO1B3, has been reported as having an influence on the efficacy of androgen deprivation therapy (ADT) in prostate cancer [7]. Thus, interference between gene polymorphisms of these androgen transporters influencing the intracellular concentration of testosterone in the tumoral cell could also play a role, at least an indirect one, in AA antitumor activity.

We thus made the hypothesis that gene polymorphisms occurring in CYP17A1, SLCO2B1, and SLCO1B3 could influence AA antitumor activity in advanced prostate cancer. Globally, these genes of potential interest cover both the target of AA and the membrane transport of AA activity modulators such as testosterone itself. This background was on the basis of a prospective, multicentric trial named Abigene conducted in consecutive patients with mCRPC. The Abigene study was initiated in 2014 and was closed for enrollment in 2017. To our knowledge, and before the current final analysis and report of the Abigene study (median patient follow-up close to six years), three publications appeared on the same topic as Abigene. They were all related to the impact of polymorphisms of genes of potential interest for AA efficacy and patient survival. Two of them were concerning the CYP17A1 gene polymorphisms [8,9], and the third one was related to the SLCO2B1 gene [10]. The Discussion section will place these publications in the context of the current results of the Abigene study.

## 2. Methods

### 2.1. Study Design and Patients

ABIGENE is a prospective, no randomized, multicenter trial covering a cohort of 148 patients between February 2014 and June 2017. The inclusion criteria were age > 18; histologically confirmed prostate adenocarcinoma; ECOG ≤ 2; evidence of metastatic disease by the presence of documented locoregional or distant metastases on CT scan of the abdomen and/or pelvis, or bone scintigraphy, patients who had disease progression after the failure of ADT. As per routine practice, AA (1000 mg) and prednisone (10 mg) were administrated daily until unacceptable toxicity or disease progression. The exclusion criteria were: patients already treated with AA, known hypersensitivity or allergy to abiraterone or any of the excipients, and at least one prior chemotherapy regimen of docetaxel for metastatic disease. Prostate-specific antigen (PSA) values have been cut off on the median, 1st quartile, and 3rd quartile. Gleason score was used to split patients with disease aggressiveness classified as low (score ≤ 6), intermediate (score = 7), or high (score ≥ 8). Informed written consent was obtained from each patient. The French Institutional Ethics Committee approved the study in 2012, and it was registered (NCT01858441).

### 2.2. Outcome and Endpoints

The primary objective of the present study was to investigate the relationships between candidate-gene polymorphisms potentially directly and indirectly related to AA pharmacodynamics by considering the rs of interest *for CYP17A1*, *SLCO2B1,* and *SLCO2B3* genes (4). Criteria for AA pharmacodynamics were: radiological response, PSA response rate, biological progression-free survival (bPFS), overall survival (OS), and toxicity. Radiologic progression-free survival (rPFS) was defined as the time elapsed between treatment initiation and radiologic progression. The rPFS rate was expressed as a 3-year progression-free survival rate. Disease progression was defined as radiographic progression in soft tissue or bone. Radiographic disease progression assessment was based on the use of computed tomography (CT) or magnetic resonance imaging (MRI) for soft-tissue lesions and defined according to modified Response Evaluation Criteria in Solid Tumors (RECIST 1.1) criteria or based on bone scan according to criteria adapted from the PCWG2 [11]. Biological response based on PSA was defined as a decrease of ≥50% in the PSA concentration from the pre-treatment baseline value, confirmed after ≥4 weeks by an additional PSA evaluation. Biological progression-free survival was assessed according to the PCWG2 criteria. PSA progression was defined as a ≥25% increase above the nadir or baseline value and when the absolute increase was ≥2 ng/mL. PSA progression was confirmed at the next study visit 3–4 weeks later. Overall survival was defined as the time between the date of treatment initiation and the date of death due to any cause. Patients showing no event (death or progression) or lost to follow-up were censored at the date they were last known to be alive. The overall survival rate was expressed as 3-year and 5-year survival rates. Treatment toxicity was evaluated with Common Terminology Criteria for Adverse Events (CTCAE 4.0). A significant adverse event was recorded as the occurrence of grade 3/4 toxicity.

### 2.3. Patient Genotyping

Genomic DNA was extracted from blood samples using the commercially available Maxwell^®^ 16 LEV Blood DNA Kit (#AS1290, Promega^©^, Madison, WI, USA). Germline DNA high-throughput genotyping was performed by MassARRAY (AGENA Bioscience^®^, San Diego, CA, USA), using the custom panel of 13 SNPs potentially related to the pharmacodynamics of AA. It comprises 9 functional SNPs of the *CYP17A* gene (allele frequency of the rare alleles > 12%) and 4 functional SNPs of the *SLCO2B1* and *SLCO1B3* genes involved in the membrane transport of testosterone and dehydroepiandrosterone (allele frequency of the rare alleles > 20%). SNPs of the *CYP17A1* gene: -34 T>C (rs743572), -362 T>C (rs2486758), 35 T>C (rs1004467), 137 G>A (rs6162), 195 C>A (rs6163), 11,994 C>A (rs4919683), 13,871 A>G (rs10883782), 15,831 G>T (rs619824), and 1243+113 T>A (rs10883783). SNPs of *SLCO2B1* gene: 312Arg>Gln (rs12422149), 3551 A>T (rs1789693), and A>G (rs1077858). SNP of *SLCO1B3* gene: 112Ser>Ala (rs4149117). All the SNPs respected the Hardy–Weinberg equilibrium. Five SNPs of the CYP17A1 gene were in pairwise linkage disequilibrium: rs4919683, rs6162, rs6163, rs619824, and rs743572 and were kept in the analysis by the investigator. Overall, the analyses were performed on 13 SNPs in the three selected genes.

### 2.4. Statistical Analysis

All 13 SNPs included in the analysis had a missing data rate of less than 10%. Dominant and recessive models were investigated to test links between SNPs and progression-free survival based on radiological evaluation and PSA measurement. For each SNP, the hazard ratio (HR) and 95% confidence interval were calculated by Cox regression for the association between the genotype and rPFS. Radiologic progression-free survival and OS were estimated using the Kaplan–Meier method. Median follow-up with a 95% confidence interval was calculated with the reverse Kaplan–Meier method. Univariate analyses were performed using the Log-rank test for survival data and using χ^2^ or Fisher’s exact test for categorical data. Variables with *p* < 0.05 in univariate analysis were introduced in the multivariate models. A genetic and clinico-pathological multivariable model was built for treatment toxicity using a backward stepwise logistic regression method. All statistical analyses were two-tailed and performed with R.4.1.1 software on Windows^®^ and LDcorSV packages [12].

## 3. Results

### 3.1. Patients’ Characteristics

A total of 137 genomic DNA samples were available for gene polymorphism analysis out of the 148 mCRPC patients included. Patients’ characteristics are summarized in Table 1. The mean age was 73 years (53 to 93 years). Performance status was 0 or 1 for 95% of patients. Tumors were mostly T3/T4 (*n* = 59, 62.77%) at diagnosis. Twenty-one patients (21%) presented with N+ stage, and 31 patients (26.5%) were de novo metastatic at inclusion. Most patients (49%) had a Gleason score of 7. Median PSA was at 32 ng/mL. The radiological response was complete for 2 patients (1.52%), partial for 8 patients (13.64%), stable for 101 patients (76.52%), and progressive disease occurred for 11 patients (8.33%). In terms of biological response, 80 patients were in complete biological response (60%), 21 patients (15.5%) in biological response, 22 patients (16.5%) in no biological response, and 11 patients in biological progression (8%). Table 2 depicts the allelic repartition for the covered SNPs of the study patients. Globally, there was almost a perfect matching between the minor allelic frequencies for all studied SNPs between the study patients and the historical European population (1000 genomes [13]).

#### 3.1.1. Association between Radiological Progression-Free Survival, SNPs, and Patients’ Characteristics

The median follow-up was 56.3 months (95% CI [52.5–61]). The 3-year and 5-year OS were 48% [41–58] and 20% [13–30], and the 3-year rPFS was 13% [8–20]. The median OS and rPFS were 35 months (95% CI [31–41]) and 15.4 months (95% CI [12.6–19.7]), respectively.

The relationship between treatment efficacy and patients’ clinico-pathological features and the 13 SNPs were analyzed by univariate analysis. As shown in Table 3, rPFS was only significantly linked with PSA (≥32 ng/mL) (HR = 1.5; IC95% [1.05–2.17]; *p* = 0.026). bPFS significantly correlated (Table 3) with rs2486758 C/C (*CYP17A1*) (HR = 4.51, IC95% [1.62–12.52]; *p* = 0.004), and PSA at baseline (≥10 ng/mL) (HR = 2.1, IC95% [1.34–3.30]; *p* = 0.001) in univariate analyses. In multivariate analysis, rs2486758 C/C and PSA (≥10 ng/mL) were associated with shorter bPFS (HR = 4.05, IC95% [1.45–11.22], *p* = 0.007 and HR = 2.07, IC95% [1.30–3.29], *p* = 0.002, respectively).

#### 3.1.2. Association between Adverse Events, SNPs, and Patients’ Characteristics

A total of 263 adverse events were identified in 137 patients, including 225 (85%) grade 1/2 and 38 (15%) grade 3/4. The main grade 3/4 toxicities were hypertension (*n* = 4; 10.7%), asthenia (*n* = 3; 8%), alkaline phosphatase (ALP) (*n* = 3; 8%) or alanine aminotransferase (AAT) (*n* = 3; 8%) increases. The relationship between adverse events, patients’ clinico-pathological features, and the 13 SNPs were analyzed by univariate analysis, as shown in Table 3. Four SNPs out of the 13 analyzed and performance status was significantly associated with toxicity: rs6162 (*CYP17A1*), rs6163 (*CYP17A1*), rs619824 (*CYP17A1*), and rs743572 (*CYP17A1*). SNPs rs6163 *(CYP17A1)*, rs6162 *(CYP17A1),* and rs619824 *(CYP17A1)* were then removed from the multivariate predictive model because they were strongly correlated. From the multivariate model, for significant toxicity, there remained two independent variables: rs743572 (*CYP17A1*) and performance status (Table 3). More precisely, rs743572 C/C *(CYP3A5)* provided an odd ratio of 3.78 (IC95% [1.42–9.75]; *p* = 0.006), and patients with performance status equal to 2 were more likely to develop grade 3/4 toxicity (OR = 4.54; IC95% [1.46–13.61]; *p* = 0.007).

## 4. Discussion

The main objective of the abigene pharmacogenetic study was to identify possible patient-related gene polymorphisms linked to AA pharmacodynamics in a consequent group of mCRPC patients. Time after the present study was initiated and before the presentation of the current data, there were two publications arising in a similar field that covered one and both concerning specifically the rs2486758. These two studies revealed the predictive value of this rs for treatment response and outcome under treatment by AA [8,9]. The present study was a strict prospective and multicentric study. It overpasses the studies by Crucitta et al. [8] and by Binder et al. [9] in terms of population size (137 patients) and duration of follow-up (median follow-up at 56.3 months). As concerns the prediction of treatment efficacy, the rs2486758 was found to have a significant link with biological 3-year progression-free survival. A higher proportion of patients with recurrence were carrying the rare C allele in a homozygous mode. Regarding the possible mechanistic explanations, such an observation can be put in line with data previously reported by Iversen and coworkers [14] showing an association between the rs2486758 (CYP17A1) genotype and the salivary concentration in 17B estradiol in healthy premenopausal women. More precisely, the presence of the rare C allele was predisposed to the highest 17B estradiol levels. As CYP17A1 acts for the production of both androgenic and estrogenic steroids, it is quite conceivable that, in the present study, patients being C/C for the rs2486758 were producing more testosterone levels than the others at the tumoral drug target level, thus ultimately explaining the relative lack of sensitivity of C/C patients to AA. Moreover, and still, from a mechanistic point of view, Manolio and coworkers [15] have shown that the rs2486758 may impact the CYP17A1 expression level, thus introducing a relative drug/target disequilibrium potentially detrimental to AA activity. Further investigations on a larger set of patients need to be conducted in order to confirm this presently reported an association between the rs2486758 and AA efficacy. Also to be taken into account in the general context of the natural evolution of prostate cancer is the link that has been suggested between the rs2486758 polymorphism and the risk of developing the disease [6]. The present data obtained for AA alone need to be confirmed in the context of drug combinations. For instance, promising results have been recently reported for advanced prostate cancer patients treated by a combination of AA and the PARP inhibitor olaparib [16].

The present study indicates that rs743572 may independently predict AA-related toxicity. Xu and coworkers reported an elevated risk of polycystic ovary syndrome in Caucasian women carrying the rs743572 C/C genotype [17]. Lower levels of androstenedione and free testosterone have been reported in rs743574-2 C/C subjects [18]. In addition, a study on 737 subjects revealed that C/C carriers exhibited significantly lower levels of 3 alpha-diol G (a reliable indicator of androgen biochemical activity in peripheral tissues) [19]. Altogether this information suggests that C/C patients may display a relatively lower capacity to in situ produce androgens and, thus, a lesser efficacy in counteracting the impact of AA in organs exposed to this drug. This may explain the greater susceptibility of rs 743574-2 C/C to AA toxic effects. This hypothesis could motivate further confirmation under specifically designed experimental conditions, especially if an increasing number of combinations based on AA are used in patients with advanced prostate cancers [20,21,22].

Interestingly a recent report by Hahn et al. [10] indicated that among a series of 79 prostate cancer patients, subjects heterozygous for rs12422149 had significantly improved progression-free survival compared with the homozygous wild-type group. The present study did not put into evidence a role for the polymorphisms of the androgen transporters genes SLCO2B1 and SLCO1B3 in AA pharmacodynamics variability. The discrepancy between the study by Hahn et al. [10] and the present one may rely on expression changes in proteins encoded by these genes impacting at different degrees on AA activity. Other biological analytical approaches, such as those based on tumor immunohistochemistry, could bring more firmly established answers on that point. In complement to the herein-considered SNPs, mention must be given to the study by Agarwal et al. [23], having shown a link between SNPs in the SULT_1_E_1_ gene and time to treatment failure for prostate cancer patients under AA.

Regarding the allelic frequencies disclosed by the study patients as confronted with data arising from 1000 genomes [13], there was an almost perfect matching in minor allelic frequencies for all covered SNPs. This means that the patients of the study and raised conclusions mostly concern the European population. Regarding the rs of interest and, more particularly, the rs2486758, 1000 Genomes data [13] reveal an underrepresentation of the C allele in the African population (4%), strongly contrasting with other populations and particularly the European one (23%). Thus, added to the fact that subjects of African origin are twice as likely than Europeans to be diagnosed with prostate cancer [24], caution must be taken when attempting to extrapolate the present data to other patient groups, particularly for patients of African origin.

The role of predictive biomarkers in treatment design and in identifying the possibility to personalize treatment has recently been underlined in patients with hormone-sensitive prostate cancer [25]. A significant place is given to biomolecular alterations, including genes affecting tumor progression. In this respect, the recent study by Swami and coworkers [26] is particularly illustrative. The authors reported that SPOP mutations (an adaptor protein of the CUL3-RBX1 E3 ubiquitin ligase complex) were associated with improved outcomes in patients under AA treatment. However, the role of the host is largely neglected. The present study fills in the gap for AA use, and this with the dual possibility of predicting treatment efficacy and identifying patients at risk for toxicity. The present data agree with recent considerations sustaining the clinical utility of germline indicators of toxicity risk [27]. As we showed that genome characteristics might help predict AA treatment efficacy and identify mCRPC patients at risk for toxicity, these conclusions agree well with the view [28] that cancer treatment should benefit patients not only in terms of improved survival but also preserving the quality of life.

## Figures and Tables

**Table 1:** Patients’ characteristics at baseline and response to treatment.

Characteristics	N° of Patients (*n* = 137)	Missing Data
Age (years) _mean [min-max]_	73 [53–93]	0 (0%)
<75 yr	76 (55.5%)	
≥75 yr	61 (44.5%)	
PSA (ng/mL) _median [Q1-Q3]_	32 [10–75]	0 (0%)
T stage _disease at diagnosis_		43 (31.4%)
T1/T2	35 (37.23%)	
T3/T4	59 (62.77%)	
N stage _disease at diagnosis_		56 (41%)
N^−^	64 (79.01%)	
N^+^	17 (20.99%)	
M _disease at diagnosis_		20 (14.5%)
Not metastatic	86 (73.5%)	
Metastatic	31 (26.5%)	
Gleason score		8 (5.8%)
Low aggressiveness (score ≤ 6)	17 (13.18%)	
Intermediate aggressiveness (score = 7)	63 (48.84%)	
High aggressiveness (score ≥ 8)	49 (37.98%)	
Neutrophil count (10^9^/L) _median [Q1-Q3]_	3.85 [1.7–5.23]	1 (0.72%)
Hemoglobin (g/dL) _median [Q1-Q3]_	13.2 [12.3–14]	1 (0.72%)
Alkaline Phosphatase (UI/L) _median [Q1-Q3]_	94.5 [70–145.25]	9 (6.5%)
Lactate dehydrogenase (IU/L) _median [Q1-Q3]_	251 [193–353.25]	13 (9.5%)
Performance status		5 (3.6%)
0	65 (49.24%)	
1	61 (46.21%)	
2	6 (4.55%)	
Radiological response		5 (3.6%)
Complete response	2 (1.52%)	
Partial response	18 (13.64%)	
Stable disease	101 (76.52%)	
Progressive disease	11 (8.33%)	
Biological response		3 (2.2%)
Biological complete response	80 (60%)	
Biological response	21 (15.5%)	
No biological response	22 (16.5%)	
Biological progression	11 (8%)	

Table keys: the radiological response was evaluated according to the RECIST1.1 criteria. The biological response was defined as a decrease of ≥50% in the PSA concentration at baseline.

**Table 2:** Summary of genotyping data for the study patients (N = 137).

Gene SNPs	*CYP17A*									*SLCO2B1*			*SLCO1B3*
	rs743572 N = 134	rs2486758 N = 135	rs1004467 N = 136	rs6162 N = 132	rs6163 N = 136	rs4919683 N = 136	rs10883782 N = 132	rs619824 N = 136	rs10883783 N = 136	rs12422149 N = 135	rs1789693 N = 136	rs1077858 N = 136	rs4149117 N = 133
Study Population	A/A (39.5%)	T/T (66%)	T/T (81%)	G/G (38.5%)	C/C (39%)	C/C (37.5%)	A/A (66%)	G/G (37%)	T/T (52%)	G/G (85%)	T/T (37%)	A/A (41%)	G/G (69%)
	A/G (44%)	C/T (31%)	C/T (17.5%)	G/A (44%)	A/C (44%)	C/A (45%)	G/A (30%)	G/T (44%)	A/T (35.5%)	G/A (13.5%)	A/T (53%)	G/A (43%)	G/T (29%)
	G/G (16.5%)	C/C (3%)	C/C (1.5%)	A/A (17.5%)	A/A (17%)	A/A (17.5%)	G/G (4%)	T/T (19%)	A/A (12.5%)	A/A (1.5%)	A/A (10%)	G/G (16%)	T/T (2%)
MAF (Allele)	0.384 (G)	0.185 (C)	0.103 (G)	0.394 (A)	0.39 (A)	0.4 (A)	0.189 (G)	0.411 (T)	0.301 (T)	0.081 (A)	0.368 (A)	0.375 (G)	0.169 (T)
Alleles (Reference Allele)	A/G/T (G)	T/C (T)	T/C (T)	G/A (A)	C/A/T (G)	C/A/T (A)	A/G (A)	T/G (T)	T/A (T)	G/A (G)	T/A/C (T)	G/A (G)	T/G/C (T)
MAF 1000 genome Europe (Allele)	0.393 (G)	0.226 (C)	0.094 (C)	0.411 (A)	0.392 (A)	0.41 (A)	0.179 (G)	0.424 (T)	0.30 (A)	0.095 (A)	0.304 (A)	0.34 (G)	0.135 (T)
SNPs Functional Impact	5 prime UTR variant	2kb upstream gene variant	Intron variant	Synonymous variant	Synonymous variant	Intergenic variant	Intergenic variant	Intergenic variant	Intron variant	Missense variant	Intron variant	Intron variant	Missense variant

Table keys: SNP = single nucleotide polymorphism; MAF = minor allele frequency.

**Table 3:** Correlations between study SNPs and progression-free survival and toxicity.

	Progression-Free Survival				
Abiraterone Acetate Treatment Predictors and Patients’ Characteristics		No Progression	Progression	HR 95%CI (Univariate Analysis)	HR 95%CI (Multivariate Analysis)
Biological Criteria					
rs2486758 *(CYP17A1)*	T/T or C/T	19 (14.5%)	112 (85.5%)	Referent	Referent
	C/C	0 (0%)	4 (100%)	4.51 (1.62–12.52, *p* = 0.004)	4.04 (1.45–11.22, *p* = 0.007)
Baseline PSA	<10 ng/mL	10 (29.41%)	24 (70.59%)	Referent	Referent
	≥10 ng/mL	9 (8.74%)	94 (91.26%)	2.1 (1.34–3.30, *p* = 0.001)	2.07 (1.30–3.29, *p* = 0.002)
Radiological Criteria					
Baseline PSA	<32 ng/mL	13 (18.84%)	56 (81.16%)	Referent	-
	≥32 ng/mL	5 (7.35%)	63 (92.65%)	1.5 (1.05–2.17, *p* = 0.026)	-
	Toxicity				
**Significant Gene Characteristics and** **Clinical Characteristics**		**Grade 1/2**	**Grade 3/4**	**OR 95%CI (Univariate Analysis)**	**OR 95%CI (Multivariate Analysis)**
rs743572 (*CYP17A1*)	T/T or T/C	199 (88.1%)	27 (11.9%)	Referent	Referent
	C/C	18 (66.7%)	9 (33.3%)	3.69 (1.45–8.88, *p* = 0.004)	3.78 (1.42–9.75, *p* = 0.006)
rs6163 (*CYP17A1*)	C/C or C/A	200 (88.1%)	27 (11.9%)	Referent	-
	A/A	22 (71.0%)	9 (29.0%)	3.03 (1.22–7.11, *p* = 0.013)	-
rs619824 (*CYP17A1*)	G/G or G/T	200 (88.1%)	27 (11.9%)	Referent	-
	T/T	25 (69.4%)	11 (30.6%)	3.26 (1.40–7.26, *p* = 0.004)	-
rs6162 (*CYP17A1*)	G/G or G/A	193 (88.5%)	25 (11.5%)	Referent	-
	A/A	23 (69.7%)	10 (30.3%)	3.36 (1.39–7.74, *p* = 0.005)	-
Performance Status	PS = 0	118 (89.4%)	14 (10.6%)	Referent	Referent
	PS = 1	77 (83.7%)	15 (16.3%)	1.64 (0.75–3.63, *p* = 0.214)	1.47 (0.62–3.46, *p* = 0.378)
	PS = 2	14 (66.7%)	7 (33.3%)	4.21 (1.4–12.08, *p* = 0.008)	4.54 (1.46–13.61, *p* = 0.007)

Table keys: Baseline PSA value at 10 ng/mL represents the first quartile, and the value of 32 ng/mL is the median value. The considered SNPs were those initially selected from univariate analysis.

## Data Availability

Individual participant data are available on reasonable request where research proposals have ethical approval. Participant data will be anonymized and only available for participants consenting to data sharing.
